# Molecular Evolution of Phototransduction Pathway Genes in Nocturnal and Diurnal Fireflies (Coleoptera: Lampyridae)

**DOI:** 10.3390/insects12060561

**Published:** 2021-06-18

**Authors:** Gavin J. Martin, Sarah E. Lower, Anton Suvorov, Seth M. Bybee

**Affiliations:** 1 Department of Biology, Brigham Young University, Provo, UT 84602, USA; antony.suvorov@gmail.com (A.S.); seth.bybee@gmail.com (S.M.B.); 2 Monte L. Bean Museum, Brigham Young University, Provo, UT 84602, USA; 3 Department of Biology, Bucknell University, Lewisburg, PA 17837, USA; sel025@bucknell.edu; 4 Department of Genetics, University of North Carolina at Chapel Hill, Chapel Hill, NC 27599, USA

**Keywords:** arrestin-2, inaC, positive selection, vision, sensory niche

## Abstract

**Simple Summary:**

Animal groups differ in the primary sense (touch, taste, smell, vision, hearing) that they use to perceive the world around them. Discovering how evolution has shaped animal senses is essential to understanding how animals interact with their environments. Fireflies are a wonderful creature to study since they use bioluminescence to find mates at night, but there are some firefly species that use other means (e.g., pheromones) to find mates and fly during the day. We examined the genes that are expressed in the eye that support sight to see if there were patterns of evolution on specific genes and different patterns of selection between day-flying non-bioluminescent and nocturnal bioluminescent fireflies. Our research is unique among beetles and is complementary to work done in flies, butterflies, and moths and allows us to better understand the boundaries of evolution on the animal visual system.

**Abstract:**

Most organisms are dependent on sensory cues from their environment for survival and reproduction. Fireflies (Coleoptera: Lampyridae) represent an ideal system for studying sensory niche adaptation due to many species relying on bioluminescent communication; as well as a diversity of ecologies. Here; using transcriptomics; we examine the phototransduction pathway in this non-model organism; and provide some of the first evidence for positive selection in the phototransduction pathway beyond opsins in beetles. Evidence for gene duplications within Lampyridae are found in *inactivation no afterpotential C* and *inactivation no* *afterpotential* *D*. We also find strong support for positive selection in *arrestin-2*; *inactivation no afterpotential D*; and *transient receptor potential-like*; with weak support for positive selection in *guanine nucleotide-binding protein* G(q) *subunit alpha* and *neither inactivation nor afterpotential C*. Taken with other recent work in flies; butterflies; and moths; this represents an exciting new avenue of study as we seek to further understand diversification and constraint on the phototransduction pathway in light of organism ecology.

## 1. Introduction

Organisms rely on sensory input from the environment to inform their basic ecology. Inputs occur across different channels, such as auditory (e.g., song), tactile (e.g., mechanosensory stimulation), gustatory (e.g., tastes), olfactory (e.g., pheromones, kairomones), and visual (e.g., bright colors, behavioral displays) channels, and are detected by different sensory structures and underlying molecular pathways. While many animals integrate input from several sensory channels depending on the circumstances, often a single channel dominates (e.g., vision in primates: [1], dragonflies and damselflies [2]), perhaps due to tradeoffs in investment in sensory structures [3,4] and the interplay of selection and constraint on the underlying molecular players (e.g., [5]). The extent to which selection and constraint play a role in adaptation to an organism’s specific sensory niche, particularly with respect to the underlying molecular mechanisms, remains a fascinating question that has been investigated mostly by work in model systems [6,7,8]. Modern sequencing technologies enable interrogation of these questions in non-model organisms, which present unique opportunities to study evolutionary forces in systems where evolutionary changes in primary sensory niche (i.e., nocturnal vs. diurnal) are known [9].

One of the best characterized signal pathways is the phototransduction (PT) pathway [8]. This is especially true in the model insect *Drosophila melanogaster* [6,10,11]. Bao and Friedrich expanded this knowledge to other model insects by examining conservation in PT genes in *Drosophila* as compared to *Tribolium*, *Anopheles*, and *Apis* [7]. This study found an increase in duplications within the higher flies (e.g., *Drosophila*) which would likely contribute to their higher photoresponse [7,12]. It has also been shown that duplication in *Drosophila* long-wavelength opsins enabled them to escape from ancestral pleiotropy [7,13,14,15,16]. However, opsins represent only one of many molecular players in the phototransduction (PT) pathway and examining the other components can help shed further light on this complex sensory system [17]. For example, recent studies in *Heliconius* butterflies have shown differences in PT gene expression beyond just the opsin genes, related to differences in visual light environment [8].

Fireflies (Coleoptera: Lampyridae) are an excellent system for studying sensory niche adaptation due to variation in their conspicuous bioluminescent mating displays. With over 2000 species distributed around the globe, variation in flash patterns across nocturnal taxa is widespread and serves in both species recognition and mate choice [18,19]. While the proximate and ultimate reasons for signal diversity in light-using species have been studied for centuries [20,21,22,23], several species have lost the ability to produce light as adults and are currently being studied. Instead of light, these day-active fireflies rely on pheromone signals to locate, recognize, and choose mates [24,25,26]. In contrast, pheromones may be relatively unimportant for nocturnal light-using species [24]. The change from nocturnal, primarily-visual, light signal use to diurnal, primarily-pheromone, signal use, makes fireflies an ideal system for testing hypotheses about the role of selection in these evolutionary transitions in sensory niches.

Previous studies in fireflies highlight the interplay of diversification and constraint in both morphological and molecular adaptations to sensory niche. Nocturnal, light-using fireflies that rely on bioluminescence for communication have larger eyes and smaller antennae than their diurnal, pheromone-using relatives [4]. This inverse relationship between eye and antenna size across sensory niches may indicate developmental constraints that limit investment in antennae at the expense of eyes, and vice versa [27]. At the molecular level, previous research has demonstrated that contrary to the insect eye bauplan, fireflies and their close relatives, along with most other beetles, have only two opsin paralogs: one which is ultraviolet sensitive and one which is long-wavelength sensitive. [28,29,30]. Sander and Hall found evidence for positive selection in both long-wavelength and ultraviolet opsin at evolutionary transitions from night to day activity [29]. Most positively selected amino acid sites in either opsin were concordant with substitutions observed in spectral shifts in other insect species. This suggests different selective pressures on the molecular level between nocturnal vs diurnal light environments. Additionally, certain fireflies have been shown to vary the expression levels of the long-wavelength opsin in correlation with peak bioluminescent signaling time [31].

**Figure 1:**
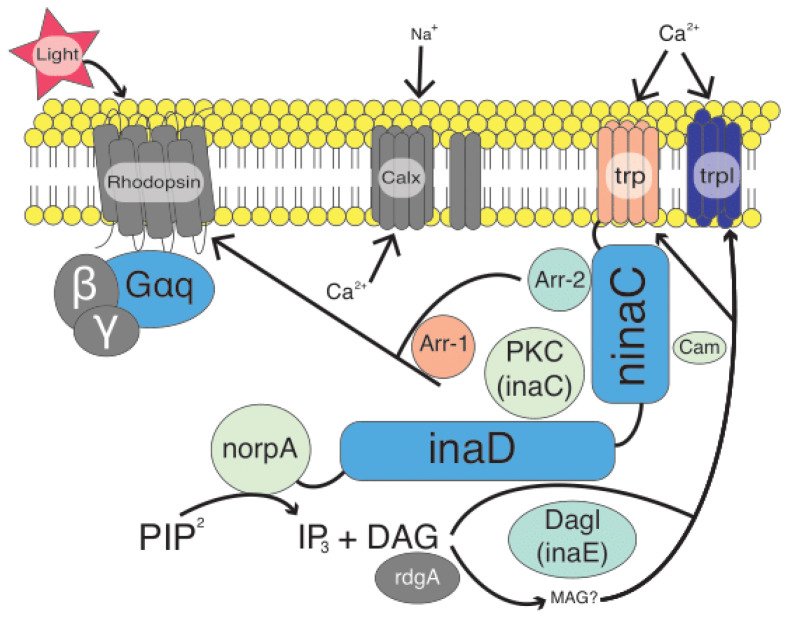
Diagram of the basic insect phototransduction pathway (modified from [6]). Components in gray represent parts of the pathway not sampled. Components in orange were sampled but were recovered in too few taxa for selection analysis. All other components are colored to reflect relative omega values as reported via the PAML analysis. Dark blue indicates high relative omega values whereas lighter blue/green indicates low relative omega values.

Vision in fireflies, like all insects, is regulated by the components of the phototransduction (PT) pathway (Figure 1). The phototransduction pathway consists of several key genes and is essentially a seven- to eight-step process that starts with light activating the insect rhodopsin and is terminated by *arrestin-1* and/or *-2* binding to the rhodopsin (for a more complete review see [8]). While most of what is known about the insect PT pathway comes from work in the dipteran fruit fly model organism, *Drosophila melanogaster,* and more recently the lepidopteran *Heliconius melpomene* [8], relatively little is known about the pathway in other insect groups, with the exception of opsins [32,33,34]. While opsin gene family expansion has been associated with selection on photoreceptor spectral sensitivity in butterflies [35], there is also evidence that opsin copy number does not correlate well with visual lifestyle in other insect groups such as dragonflies [36]. Opsins have also been well studied in fireflies [28,29]. Further, exploring the full phototransduction pathway in fireflies would provide one of the first studies of this pathway with respect to signal niche adaptation and especially potential for selection in beetles.

With growing genomic tools for non-model organisms, including fireflies [29,37], it is possible to examine the entire PT pathway and test hypotheses regarding the visual evolution of these organisms. Here, we search a total of 32 both publicly available (10 transcriptomes) and novel unpublished (22 transcriptomes) firefly transcriptomes representing 20 species in total to address the following objectives: (1) Identify genes involved in phototransduction in firefly transcriptomes; (2) Test hypotheses of selection related to sensory niche adaptation (nocturnal vs diurnal); and (3) as a secondary focus, explore gene duplication and loss among fireflies and as compared to other, model, insects (i.e., *Drosophila* and *Tribolium*). 

## 2. Materials and Methods

### 2.1. Sampling and Sequencing

**Figure 2:**
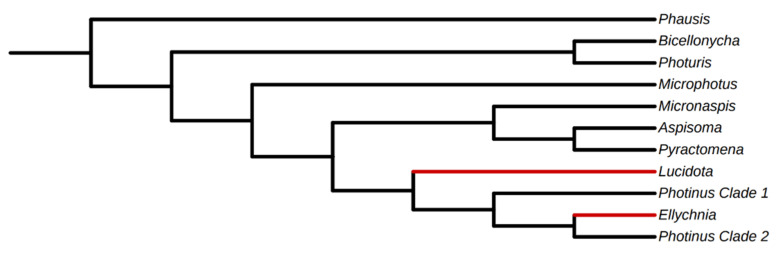
Cladogram showing generic relationships of sampled taxa. Relationships modified from [29,38]. Taxa in red indicate diurnal lineages.

Because genome assemblies are available for only three firefly species (all lighted taxa), only one of which is closely related to a diurnal species with existing -omic data, PT genes were identified from previously published [29] and new [28] transcriptome assemblies (see Figure 2 for cladogram of generic relationships among sampled taxa) to capture taxonomic and ecological breadth. It should be noted that while [28] used transcriptome sequences to study opsin evolution, at the time only the opsin gene sequences were reported. Raw reads and transcriptome assemblies for the 22 libraries from [28] are reported here for the first time.

RNA was extracted from several body parts (i.e., head only, abdomen/light organ only, and whole body). As such, while there are 32 RNA-seq libraries in the study, these represent 20 taxa (Supplementary Table S1; https://doi.org/10.5061/dryad.mkkwh70vq) (accessed on 8 June 2021). Both head and body tissue for transcriptome assembly was prepped separately for the following taxa: *Micronaspis floridana* Green, *Pyractomena dispersa* Green, *P. pyralis* Linnaeus (see [28]). Total RNA was extracted from each taxon using NucleoSpin columns (Clontech, Mountain View, CA, USA) and reverse-transcribed into cDNA libraries using the Illumina TruSeq RNA v2 sample preparation that both generates and amplifies full-length cDNAs. Prepped mRNA libraries were sequenced either on an GaIIX using 72 paired-end reads (RCO_011–RCO_025) by the DNA Sequencing Center at Brigham Young University, Provo, UT, USA or on an Illumina HiSeq 2000 utilizing 101-cycle paired-end reads (RCO_27–RCO_36) by the Microarray and Genomic Analysis Core Facility at the Huntsman Cancer Institute at the University of Utah, Salt Lake City, UT, USA. Voucher specimens are deposited at BYU (for those with RCO numbers) or UGA (for those with KSH numbers). To aid in future identification, the COI sequences are also available on genbank (Supplementary Table S2). The transcriptomic data allows us to combine current data, as well as gain a knowledge of what parts of the phototransduction pathway are actually expressed in the firefly eye. 

### 2.2. Transcriptome Assembly and Annotation

Quality control, assembly, and transcriptome searches were performed using a pipeline constructed from existing computational tools after [36] to facilitate downstream evolutionary analyses. In short, RNA-seq reads were trimmed using the Mott algorithm implemented in PoPoolation version 1.2.2 [39], with a minimum read length = 40 and quality threshold = 20. The de novo assembly of the transcriptome contigs was carried out using Trinity version 2.0.6 [40] under the default parameters. Assembled transcriptomes were then annotated using the trinotate release 2013-08-26 pipeline. Detection and filtering for putative phototransduction genes were performed against a database comprised of insect visual phototransduction homologous groups taken from OrthoDB database [41]. Recovered insect sequences from the PT pathway were then aligned using MAFFT v. 7.407 [42] and converted into a profile hidden Markov model (pHMM) database using hmmbuild program of HMMER 3.1b1 [43]. Using this database, we screened TransDecoder-predicted proteomes for genes known from the PT pathway against pHMM database using hmmscan with an e-value cutoff of 10^−5^. Additionally, we used PIA [44] with default parameters to identify phototransduction genes that may be missed by the HMMER search using the raw Trinity transcriptome assemblies. This resulted in approximately 80 gene sequences. All redundant (identical) phototransduction gene sequences identified by both approaches were removed using CD-HIT [45,46]. After all putative hits were compiled, sequences were screened for any false positives, including sequences coding for other genes, or duplicate reads not the result of biology, but rather read coverage. Sequences were aligned via MAFFT with the outgroup beetle *Tribolium castaneum* to remove sequences with >98% similarity. Each unique sequence was then Blast searched using megablast against the NCBI database to confirm gene identity. Only those genes listed in the KEGG phototransduction pathway were included in downstream analysis (Table 1). Assemblies were assessed for quality and completeness using BUSCO v. 3 [47], comparing against the endopterygota_odb9 database.

### 2.3. Analyses of Positive Selection

Sequences for each gene were aligned independently in MAFFT using the outgroup taxon *Tribolium castaneum* and the default automatic alignment option (for alignments see dryad repository). Each subsequent alignment was used for Maximum Likelihood tree inference implemented within IQ-Tree v. 1.6.8 [48]. Tests for positive selection in each gene were performed in PAML v. 4.9 [49]. Using the branch-specific test, the free-ratios model (Ha; model = 2, nsites = 0) was tested against the branch model (Ho; model = 0, nsites = 0). The log likelihood of each model was compared with the likelihood ratio test (LRT) using *X*2 distributions (3.84 at *p*-value = 0.05) with appropriate degrees of freedom.

Inference of positive selection has been shown to be greatly influenced by the accuracy of MSA [50]. In particular the inference of selection obtained on MSAs produced by MAFFT shows overconfidence in identification of positive selection (i.e., increased rates of false positive results). In order to mitigate that problem, we implemented a Bayesian approach utilized in BAli-Phy version 3.4.1 [51], that essentially jointly estimates MSAs and positive selection (in our case branch-specific) and exhibits superior accuracy [50]. To run analyses of branch positive selection in BAli-Phy, for each gene we used the corresponding tree estimated by ML initializing 3 independent MCMC chains with 3000 iterations each as in [50]. MSA was sampled every 5th iteration. The seemingly low number of iterations is explained by the fact that at each MCMC draw, BAli-Phy updates multiple parameters compared to other MCMC software where only one parameter is updated per iteration. In order to improve our estimator of positive selection we used the Rao-Blackwellization technique, i.e., taking a conditional expectation of the current estimator [50].

The results of 3 runs were pulled together discarding 15% burn-in for each gene. Then Bayes factors (BF) were calculated to assess support for positive selection. We followed a scoring scheme proposed by [52], where BF > 20 exhibits strong support for positive selection, 20 < BF < 3 exhibits “positive” support and BF < 3 is “not worth more than a bare mention”. 

To further explore positive selection in these diurnal lineages, we also ran branch-site tests to detect positive selection in specific amino acid sites (Ho: ⍵ = fixed at 1; Ha: ⍵ = variable) [53]. The models were tested as above using the LRT. 

## 3. Results

### 3.1. Transcriptome Assemblies Recover Most Conserved Genes

To examine patterns of gene duplication and test for selection in the PT pathway across firefly species, we compiled transcriptome assemblies and gene trees from published [28,29] and new datasets. Transcriptomes varied in quality, generally according to input tissue type (Supplementary Table S1). Transcriptome assemblies captured the majority of conserved genes as full-length transcripts—mean BUSCO completeness across assemblies was 76% (range: 40–95%), the mean N50 was 2096 bp, and the mean maximum contig length was 17,138 bp. Total number of contigs varied from 19,676 to 77,811 (Supplementary Table S1).

### 3.2. Ortholog Search Strategy Identifies Phototransduction Genes

Ortholog search, followed by manual curation of results, identified twelve genes in the PT pathway across the transcriptomes in this dataset, including *arrestin-1* (*arr1*), *arrestin-2* (*arr2*), *calmodulin* (*Cam*), *guanine nucleotide-binding protein G(q) subunit alpha* (*Gq*), *G protein-coupled receptor kinase 1* (*Gprk1*), *inactivation no afterpotential C* (*inaC*), *inactivation no afterpotential D* (*inaD*), *inactivation no afterpotential E* (*inaE*), *neither inactivation nor afterpotential C* (*ninaC*), *no receptor potential A* (*norpA*), *transient receptor potential* (*trp*), and *transient receptor potential-like* (*trpl*). Unfortunately, *arr1, Cam,* and *trp* were not recovered from any diurnal species. Therefore, we were unable to test these genes further. Because, to date, no sampled insect has been conclusively shown to be missing *arr1*, *Cam*, or *trp*, the absence of these genes in our data is likely due to low/no expression in the tissues sampled or insufficient sequencing depth. While not every gene was found in every species, putative duplications of various genes were found in several species: *Photinus pyralis* (*inaC*), *Photinus macdermotti* (*inaC*), *Lucidota atra* (*inaC*), *Phausis reticulata* (*inaC*), *Bicellonycha wickershamorum* (*inaC*), and *Photinus carolinus* (*inaD;* however, see below); see Table 1, Supplementary Table S3 and supplemental trees. The putative duplicate of *inaC* is present in the *Photinus pyralis* genome [37], the only species with a genome that overlaps with the taxa sampled in this study. The putative duplicate was on a different linkage group (*inaC*: LG1, LOC116181404, *inaC* copy: LG6, LOC116173989). However, the ortholog of the putative duplicate of *inaD* within *Photinus carolinus* does not seem to be present in the *P. pyralis* genome, and requires further confirmation. 

### 3.3. Analysis for Positive Selection Identifies Genes under Positive Selection

#### 3.3.1. PAML

Tests for positive selection were performed in PAML [49] on two sets of trees, resulting from two different approaches to alignment. First, PAML was run on the original MAFFT alignment using *Tribolium castaneum* as the outgroup for each gene. The branch model test (model = 0, nsites = 0) for the null hypothesis showed elevated omega values (average ⍵ across all genes 0.081) for *Gq* (⍵ = 0.114), *inaD* (⍵ = 0.114), *ninaC* (⍵ = 0.110)*,* and *trpl* (⍵ = 0.230). This analysis also supported the evidence for positive selection (likelihood ratio test statistic (LRT) > 3.84 at *p*-value cutoff 0.05) in *arr2* (LRT = 43.59), *inaC* (LRT = 64.35), *inaD* (LRT = 28.04), *ninaC* (LRT = 43.76), and *trpl* (LRT = 16.07) when the alternative hypothesis (model = 2, nsites = 0) was selection in diurnal lineages. 

In addition to branch model tests, branch-site tests were performed on the original MAFFT alignment. Only *Gq* and *trpl* passed the LRT statistic suggesting specific amino acid sites (sites 2, 37, 38, 39, 79, 80, 97, 98, 101; and 967, 1204, 1215, 1225 respectively) under positive selection in diurnal lineages (Supplementary Table S4). 

#### 3.3.2. BAli-Phy

Additionally, PAML was run on the alignment resulting from BAli-Phy [51,54] to account for potential overestimation of positive selection in the MAFFT alignment [50]. This analysis resulted in elevated omega values (average ⍵ across all genes 0.049), *inaD* (⍵ = 0.092), *ninaC* (⍵ = 0.109)*,* and *trpl* (⍵ = 0.082). The PAML analysis of the BAli-Phy alignment also resulted in support for evidence of positive selection in *arr2* (LRT = 56.28), *Gq* (LRT = 5.02), *inaD* (LRT = 22.30), *ninaC* (LRT = 45.10), and *trpl* (LRT = 114.44); (Table 2). Consistent between the two PAML results *arr2*, *inaD*, *ninaC*, and *trpl* were identified as possibly evolving under positive selection. 

**Figure 3:**
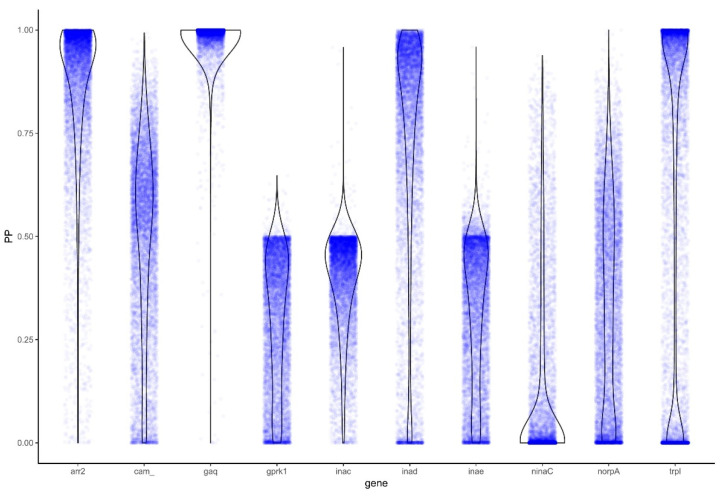
Distributions of the posterior probability (PP) of positive selection across phototransduction genes. Violin plots represent estimated kernel density MCMC posterior probability of positive selection (blue dots).

To further explore genes identified by PAML to be under positive selection, we also tested them for selective-constraint using BAli-Phy by examining the Bayes Factor (BF). The genes *arr2* (BF: 9.5), *Gq* (82.28), *inaD* (5.13), and *trpl* (8.59) were again identified as evolving under positive selection (Figure 3; Table 2). 

Given these results, there is strong evidence in all three analyses for *arr2*, *inaD*, and *trpl* being under positive selection in diurnal lineages, with additional support in two analyses for positive selection in *ninaC* and *Gq* (Table 2).

## 4. Discussion

The insect phototransduction cascade is most well-known and studied in the model insects *Drosophila* and *Heliconius* (reviewed in [6,7,8]). Here we increase the knowledge of this cascade system by exploring the PT pathway in fireflies as examined in both novel unpublished and publicly available transcriptome data. We further explore these data by looking for patterns of selection that are potentially related to the signaling ecology of nocturnal vs diurnal fireflies. 

Despite the notorious incompleteness of transcriptome data due to tissue-specific expression, we were able to find evidence for potential duplications of genes in the PT pathway in fireflies. Duplication rates of phototransduction genes have been shown to be higher in arthropods and related groups as opposed to other metazoan organisms [54]. These duplications have been demonstrated in several gene families such as TRP, r-opsin (rhabdomeric), and arrestin among others [8,54]. Here we add to the known duplication events in insects by showing evidence for duplications of *inaC* and *inaD* within fireflies. The low number of duplicates in the firefly PT pathway is in line with what has been reported in other insects (~15% in *Drosophila* compared to ~1.3% in *Tribolium*) [7].

In insect phototransduction, *inaD* acts as a scaffold protein that is central to a group of proteins commonly known as the signalplex [55,56,57,58,59]. This signalplex is composed primarily of *inaD*, *trp*, *norpA*, and *inaC*, also found in one of our analyses to be under positive selection [6]. If *inaD* is lost or disrupted, it causes a downstream breakup of the signalplex [60]. Given the critical nature of *inaD*, duplications in this gene could provide redundancy or allow for differential expression. Additionally, *inaD* conservation has been shown to contribute to the remarkably fast photoresponse in the higher Diptera [61,62]. One hypothesis linking duplication to ecology is that duplicates in the signalplex could allow for amino acid changes to facilitate an increase in photoresponse [7]. Whether fireflies have an increased photoresponse compared to other beetles remains unknown. Further, *inaD* stability is dependent upon *trp* [6,63,64]. Further upstream in the cascade *Gq* plays a role in the portion of the cascade that leads to the production of DAG and MAG [65] which are hypothesized to play a critical role in activation of *trp* and *trpl* [8,66]. 

The interaction between *trp* and *trpl* in organisms which inhabit low-light vs. high-light environments has received much attention recently. It appears that one or the other can be up- or down-regulated depending on the dominant light environment. In *Drosophila melanogaster* (active primarily during the day) *trp* is not only the more abundant channel, but also flies act blindly with mutated *trp* [67]. In cockroaches (primarily nocturnal) the opposite was shown, *trpl* was the more abundant channel by far [68]. Recently, Macias-Munoz and colleagues [8] have shown an interesting relationship within the Lepidoptera. The day active *Heliconius melpomene* showed no differentiation in *trp* vs. *trpl* channels, however the night-active *Manduca sexta* showed lower relative expression of *trp*, showing a similar pattern to that of the cockroaches [8]. Here, we show strong support for the hypothesis that *trpl* is under more positive selection in the diurnal fireflies *Ellychnia* and *Lucidota.* Phrased another way, *trpl* is likely under purifying selection in the nocturnal firefly lineages, showing the consistent importance of *trpl* for nocturnal lineages. 

*InaD* also forms a protein complex with *ninaC* (*ninaC* was also recovered in two of the three tests for positive selection), each of them binding *Cam*. This complex has been shown to mediate Ca^2+^ movement, thus greatly increasing the efficiency of arrestin [69,70]. In order for arrestin movement to take place, arrestins must bind to phosphoinositides, which is thought to be mediated via *ninaC* ([71], however see [72]). 

There is also strong support for the detection of positive selection in *arr2* in diurnal species of fireflies. The arrestin gene family is well known for mediation in many G protein-coupled receptor signaling cascades [73]. In these systems, its purpose is to arrest or stop signaling [74,75]. While there are four known arrestin genes in mammalian systems [76], only two, *arrestin-1* and *arrestin-2,* have been found in *Drosophila* and *Heliconius* [8,77,78,79,80]. Both arrestin genes were expressed in our Lampyridae taxa, however *arrestin-1* was only found in a handful of taxa, and not recovered in either of the diurnal species included. In Lepidoptera, *arr2* is more highly expressed than *arr1. Arr2* does not reside in the rhabdomere (where phototransduction takes place) but is trafficked into the rhabdomere after phototransduction (see above). 

Our results are compelling and demonstrate that fireflies, and likely bioluminescent beetles in general, are a strong system to study the relationship between ecology (e.g., signaling and activity times) and molecular evolution (e.g., selection and gene duplication). We provide the first significant steps in this direction and provide evidence for selection in the broader firefly visual system. In the future, using genomes from bioluminescent beetles to more deeply investigate the evolution of genes involved in the PT pathway by exploring synteny among these genes and more extensively exploring orthology among duplicates would provide deeper insight into how ecology may be driving and even maintaining genetic diversity at the genomic level. Here, our research was constrained as only one high-quality genome overlapped with the transcriptomes for the species we investigated. 

We also identified several areas where functional analyses will further aid in the discussion of selection in the phototransduction pathway. For example in *guanine nucleotide-binding protein G(q) subunit alpha* multiple amino acid sites were found to have evidence for positive selection including a stretch from residue 37–39, which in *Drosophila* is the beginning of the G1 motif and extremely close to a nucleotide-binding region (AA40-47). Evidence for positive selection at specific sites in *transient receptor potential like* does not correspond with known functional regions in *Drosophila* (Supplementary Table S4).

Additionally, an in-depth quantification and qualification of PT genes across multiple firefly genomes, as well as expression data from throughout the diel changes period to compare potential differences of PT gene expression, is needed. Such experiments would also provide insight into dimorphic gene expression patterns as has been found in one species of firefly [31]. Firefly modes of communication have been described under various systems [81,82], however, most recently firefly communication modes were simplified into four groups: pheromone use only, continuous glow + pheromone, short or long flashes, and pheromone use + weak, daylight glow [83]. Sampling from these communication modes would allow for a finer scale look at the evolution of phototransduction genes.

Finally, tests for positive selection are subject to false positives [84] and functional follow-up studies are the necessary next step to further investigate the results presented here. While functional studies are currently infeasible or impossible for most firefly species because they have a long life cycle and cannot be reliably cultured in the lab, recent advances in applying CRISPR to *Aquatica lateralis*, a lab-culturable species from Japan, are promising [85]. Additionally, protein function can be further studied in vitro, in tissue culture, and in transgenic *Drosophila*. All of these offer exciting future avenues for further study. Understanding the protein function will help in the characterization of the *inaC* duplicate. While the duplicate was found mostly in bioluminescent species, it was also found in the non-bioluminescent *Lucidota atra*. Therefore it seems likely that the duplicate may serve more of a role in increasing overall visual acuity [86] as opposed to having a specific function in the transitions in sensory niche. 

## 5. Conclusions

This study represents one of the first attempts to assess positive selection in the phototransduction genes in a non-model insect beyond the r-opsin gene family. *Arr2*, *inaD,* and *trpl* represent critical components of the insect phototransduction pathway. All three are associated with light-dependent interactions within the eye. While sexual communication in many fireflies is critically dependent on vision, bioluminescent communication tends to take place beginning in the dusk hours and continuing into the night. In this dark environment, regardless of sexual activity, there are simply fewer photons reaching the firefly eye, meaning the PT pathway is not as active. For those lineages (*Ellychnia* and *Lucidota*) active during the day, and therefore more dependent on light for survival and reproduction, it is intriguing that these light-activated genes have support for evolving under positive selection, and are therefore diverging from those in the conserved, nocturnal sensory niche. As we investigate the variation in this system, both with a larger taxon sampling, and with more complete genomic resources, our understanding of insect vision will come into sharper focus. While this study adds an important component in insect visual research of non-model organisms, more remains to be addressed. As it stands, we have successfully identified several genes that are strong candidates for further positive selection studies. In the case of *inaC*, we have also identified a tantalizing area of study into the loss of certain phototransduction genes across the beetle tree of life. Further study will surely help our understanding of positive selection in terms of adaptation to differing sensory niches.

## Figures and Tables

**Table 1:** Presence/absence data for genes sampled in the phototransduction pathway. Gray box indicates presence. * indicates presence of duplication in *Photinus carolinus* only.

Species	*arr1*	*arr2*	*Cam*	*Gq*	*Gprk1*	*inaC*	*inaC Copy*	*inaD*	*inaE*	*ninaC*	*norpA*	*trp*	*trpl*
*Aspisoma* sp.													
*Bicellonycha wickershamorum*													
*Ellychnia* sp.													
*Lucidota atra*													
*Micronaspis floridana*													
*Microphotus* sp.													
*Phausis reticulata*													
*Photinus australis*													
*Photinus carolinus*								*					
*Photinus macdermotti*													
*Photinus marginellus*													
*Photinus pyralis*													
*Photinus scintillans*													
*Photuris* “A”													
*Photuris frontalis*													
*Photuris* sp.													
*Photuris* sp. 1													
*Photuris* sp. 2 larva													
*Pyractomena borealis*													
*Pyractomena dispersa*													

**Table 2:** Summary of positive selection analyses carried out in PAML and BAli-Phy. P value for lrt significance is 0.05. Level of support for Bayes Factor is after [52].

Summary of Positive Selection Analyses	PAML														BAli-Phy	
	MAFFT Alignment							Bali-Phy Alignment								
	Model	lnl	lrt	np	df	*p*-Value	Omega	Model	lnl	lrt	np	df	*p*-Value	Omega	Bayes Factor	Level of Support
***arr2***	null	−3504.00804	43.58565	19	1	<0.00001	0.0545	null	−3447.79062	56.286878	19	1	<0.00001	0.0459	9.502073	Positive
	alt	−3482.21521		20				alt	−3419.64718		20					
***Gq***	null	−6134.51482	0.050698	35	1	0.82202	0.1139	null	−5227.9707	5.017252	34	1	0.0251	0.0305	82.27678	Strong
	alt	−6134.48947		36				alt	−5225.46207		35					
***Gprk1***	null	−9493.07143	1.05478	45	1	0.30443	0.0087	null	−9526.73339	0.411348	41	1	0.52131	0.0081	0.623359	None
	alt	−9492.54404		46				alt	−9526.52772		42					
***inaC***	null	−17053.9096	64.3459	61	1	<0.00001	0.0186	null	−16617.8413	0.06558	58	1	0.79788	0.013	0.778239	None
	alt	−17021.7366		62				alt	−16617.8085		59					
***inaD***	null	−19140.0313	28.04327	45	1	<0.00001	0.1139	null	−18588.4152	22.296904	43	1	<0.00001	0.0921	5.128159	Positive
	alt	−19126.0097		46				alt	−18577.2668		44					
***inaE***	null	−21162.9887	0.938762	45	1	0.33261	0.0576	null	−20557.5219	0.925638	42	1	0.33601	0.0429	0.673093	None
	alt	−21162.5193		46				alt	−20557.0591		43					
***ninaC***	null	−17058.6637	43.75643	15	1	<0.00001	0.1104	null	−16930.1126	45.098156	15	1	<0.00001	0.1092	0.990066	None
	alt	−17036.7855		16				alt	−16907.5635		16					
***norpA***	null	−19292.2825	0.01304	43	1	0.90909	0.0226	null	−18587.9541	3.361894	42	1	0.06673	0.0145	1.041881	None
	alt	−19292.276		44				alt	−18586.2732		43					
***trpl***	null	−23948.4017	16.06823	33	1	0.000061	0.2298	null	−27362.9713	114.4442	33	1	<0.00001	0.0821	8.59116	Positive
	alt	−23940.3676		34				alt	−27305.7492		34					

## Data Availability

The data sets and trees supporting the results of this article are available in the datadryad repository, https://doi.org/10.5061/dryad.mkkwh70vq (accessed on 7 June 2021). Raw reads generated in this study are available through BioProject PRJNA737479. COX1 sequences from as-yet-unidentified species are available on NCBI (accessions: MZ394513–MZ394519).

## Supplementary Materials

The following are available online at https://www.mdpi.com/article/10.3390/insects12060561/s1, Table S1, Assembly summary statistics for transcriptomes, Table S2, SRA and NCBI accession numbers for new data, Table S3, Presence/absence data for genes sampled in the PT pathway, & Table S4, Summary of branch-site analyses.
